# Influence of Environment and Rootstock On the Rhizosphere Bacterial Communities in Four Vineyards of the Douro Demarcated Region

**DOI:** 10.1007/s00248-026-02798-z

**Published:** 2026-06-11

**Authors:** João Prada, Leandro Pereira-Dias, João A. Santos, Conceição Santos

**Affiliations:** 1 Centre for the Research and Technology of Agroenvironmental and Biological Sciences, CITAB, University of Trás-Os-Montes and Alto Douro, UTAD, Vila Real, Portugal; 2 LAQV-REQUIMTE, Faculdade de Ciências da Universidade Do Porto, Porto, Portugal; 3 iB2, Departamento de Biologia, Faculdade de Ciências da Universidade Do Porto, Porto, Portugal; 4 Fundação Côa Parque, Vila Nova de Foz Côa, Portugal; 5 Instituto de Conservación y Mejora de La Agrodiversidad Valenciana, Universitat Politècnica de València, Valencia, Spain

**Keywords:** Climate change, Douro Demarcated Region, Microbiome, Rootstock, Viticulture

## Abstract

The Douro Demarcated Region (DDR) is a worldwide acknowledged winemaking region. Climate change is threatening the sector, as climatic shifts are expected. This work analyzed environmental and genetic traits modulating the rhizobiome of four vineyards in the DDR, focusing on understanding the hierarchy of ecological conductors for these communities. These vineyards’ *terroir* was environmentally, genetically, and culturally characterized. Rhizosphere bacterial community composition was analyzed using 16S metabarcoding from soil samples collected between July 2022 and January 2024. Results support the hypothesis of an ecological profiling hierarchy. The soil physicochemical properties likely acted as a primary environmental modulator, determining the composition of the bacterial microbiome and contributing to the diversity and richness of the bacterial communities. The major drivers among the soil’s physiochemistry were organic/inorganic profile, mainly influenced by organic matter content and pH. Rootstock genotype appears to exert a secondary selection on the microbiome, focusing on the microorganisms’ functional traits. The 1103-P rootstock positively influenced the abundance of copiotrophic bacteria, compared to the R110, demonstrating that the first recruits a more versatile, exploratory, and expansionist microbiome, whilst the second focuses on attracting a highly specialized community, more focused on maximizing energy gathering and optimizing resource use. This work demonstrates that the microbial *terroir* is a result of multiple factors, promoting abiotic modulation and host-mediated selection, which establishes a very specific community for each scenario. The assessment of these holistic dynamics is fundamental to establishing a baseline for future precision viticulture strategies, namely bio-inoculants, to support and promote a better grapevine adaptation to climate change.

**Supplementary Information:**

The online version contains supplementary material available at 10.1007/s00248-026-02798-z.

## Introduction

Viticulture is one of the most dynamic and culturally significant socioeconomic activities worldwide. It not only produces high-value products but also plays a vital role in the socio-economic development and cultural identity of many regions, particularly across the Mediterranean basin. In 2023, global wine production generated approximately € 36 billion in revenue, making it one of the leading sectors in the agricultural economy [91]. In Portugal, the Douro Demarcated Region (DDR) stands out as the origin of the renowned Porto wine. This region encompasses a diversity of *terroirs* shaped by a combination of climatic, soil, geographic, and cultural factors [80]. The role of soil microbiomes in defining terroirs is increasingly being recognized [1]. Microbial communities play a fundamental role in grapevine physiological processes, including tolerance to biotic and abiotic stresses, and significantly influence grape production, thereby affecting the composition and quality of the final product [81]. Soil microbiomes are typically highly diverse [82] and are influenced by complex ecological factors, such as temperature and precipitation patterns, nutrient availability, and landscape [2, 3]. These factors act as primary environmental filters, modulating the microbiomes’ regional pool [4, 5, 83–85].

Genetic factors, such as grapevine variety and rootstock, are also influential towards the microbiome communities, exerting a secondary selective influence and shaping biochemical interactions with specific microorganisms [6, 7, 86–88]. These functions ultimately impact root development, plant growth, yield, susceptibility to pests and diseases, and ultimately shape product quality [8, 82, 89, 90].

Despite the recognition of the influence of these isolated factors, there is a gap in knowledge regarding the hierarchy and interaction between these drivers in heterogeneous fields and under climatic pressure. Usually, past works focus on unique factors to understand their influence on microbiomes, leaving unanswered the influence of the interaction between biotic and abiotic factors on microbiome shaping.

Our work aims to understand the multi-factor conditions that shape microbiome diversity in each *terroir* of the DDR. We hypothesize that macro-environmental factors, such as the soil’s physicochemical properties, are the primary determinants of the bacterial community in the vineyards’ rhizosphere, and that the rootstock’s genotype acts as a specific functional filter, attracting different types of bacteria, depending on the selected genotype. These static factors could exert a stronger selective pressure on the microbiome than seasonal fluctuations.

With this understanding, we will comprehend the major influential factors concerning bacterial microbiome variability and whether they promote/restrict bacterial diversity, and how they modulate functionality. Furthermore, it is of major importance to understand which functional patterns are influenced and, therefore, comprehend which are prioritized, to ultimately assess the holistic impacts of environmental and genetic factors on the grapevine’s soil microbiome and on its physiological activity.

The characterization of these specific microbiomes will support future works in identifying potential bacterial targets, which could be explored in viticultural practices, such as the development of bio-inoculants tailored to specific *terroirs* [4, 5, 9, 10].

## Materials and Methods

### Vineyards' Genetic and Terroir Characterization

**Fig. 1:**
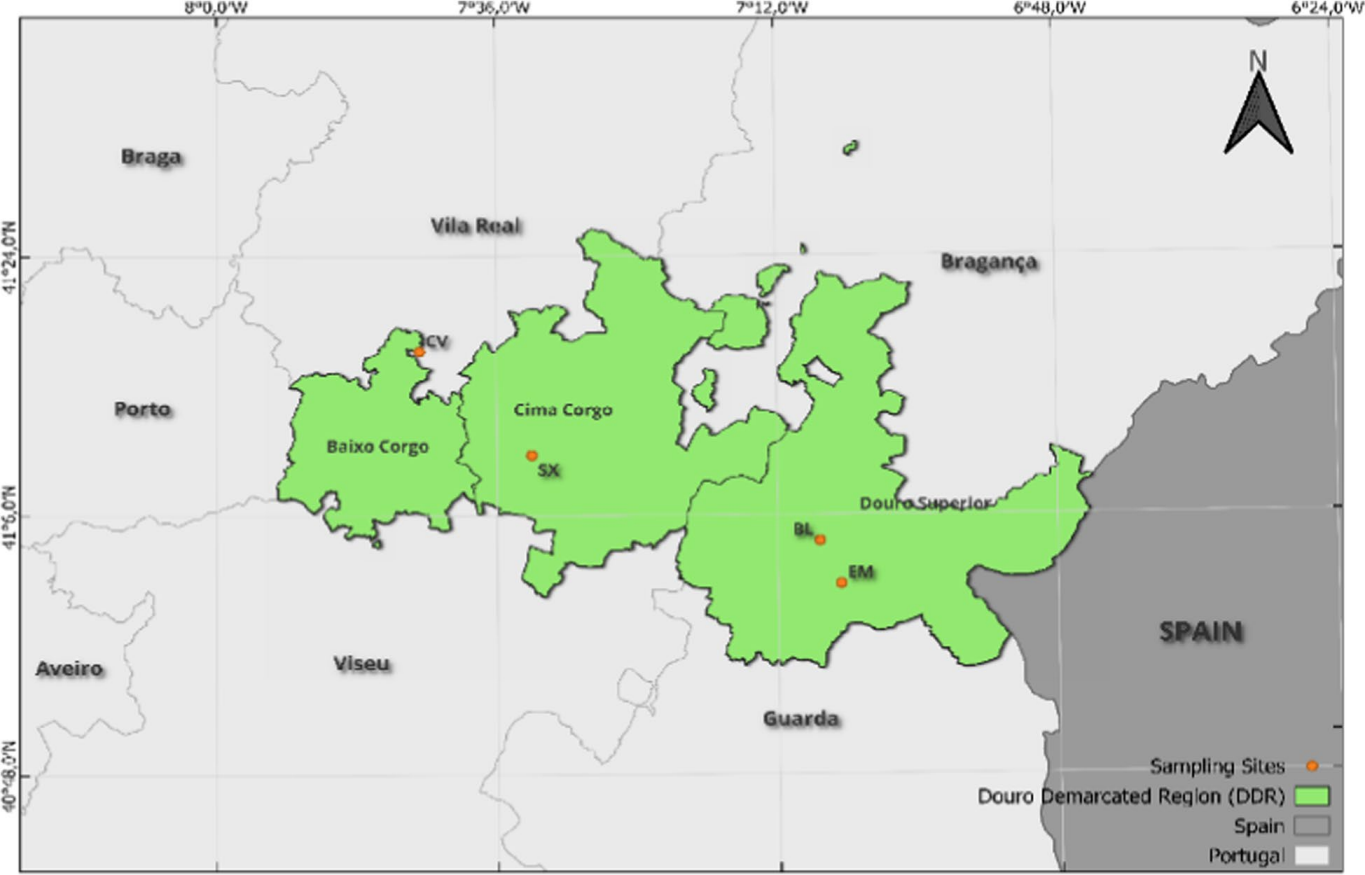
Location of the sampling sites (vineyards; orange dots) within the Douro Demarcated Region (DDR): Quinta do Cavernelho (CV), Baixo Corgo; Quinta do Seixo (SX), Cima Corgo; Quinta Belém (BL), Douro Superior; and Quinta da Ervamoira (EM), Douro Superior

To capture the spatial variability of terroir within the DDR, four vineyards from different subregions were selected for study. These included Quinta do Cavernelho (CV) in Baixo Corgo (41°17′38.62″ N, 7°43′13.06″ W), Quinta do Seixo (SX) in Cima Corgo (41°09′59.37″ N, 7°33′9.82″ W), and Quinta Belém (BL; 41°06′12.0″ N, 7°07′27.1″ W) together with Quinta da Ervamoira (EM; 41°01′23.3″ N, 7°06′51.8″ W) in Douro Superior. Their locations are illustrated in Fig. 1.

Terroir characterization was based on a questionnaire administered at the beginning of the sampling campaign (2022) to the production manager of each vineyard. The questionnaire covered several aspects of vineyard characteristics and management practices (Table 1).

**Table 1:** Environmental, genetic, and cultural properties of the vineyards in the study

	**Vineyard**	**CV**	**SX**	**BL**	**EM**
**Environmental**	**Altitude (m)**	465	215	340	120
	**Area of the vineyard (ha)**	0.57	0.75	1.5	2.9
	**Inter-row Spacing (m)**	2.2	2.2	2.2	2.2
	**Inter-plant Spacing (m)**	1.2	1.2	1.2	1.2
	**Landscape**	Highland	Scarp	Highland	Lowland
**Genetic**	**Vine variety**	Tinta*–*Roriz	Tinta*–*Roriz	Tinta*–*Roriz	Tinta*–*Roriz
	**Grape colour**	Red	Red	Red	Red
	**Age of the vineyard (years)**	25	15	12	47
	**Rootstock**	1103-P	R110	1103-P	R110
**Cultural**	**Production system**	Integrated	Integrated	Integrated	Integrated
	**Irrigation**	No	No	No	Yes (Summer)
	**Cover Crops**	No	No	No	No
	**Soil fertilization**	No	No	Organic matter	Organic matter

The vineyards comprising this work are planted with the Tinta Roriz, also known as Tempranillo, cultivar (*Vitis vinifera* L.), a vigorous, early ripening red grape Iberian variety, known for being sensitive to severe drought and heat stress [11]. The Tinta Roriz vineyards were implemented with 1103-P and R110 rootstocks (2 vineyards per rootstock), both being *V. berlandieri* x *V. rupestris* hybrids. The 1103-P is characterized by having high vigor and deep rooting systems that actively maintain water flux and support the grapevine’s physiology [12, 13], whilst the R110 is tailored for poor, rocky soils, having a conservative water-saving strategy [12].

### Soil Physicochemical Analysis

To perform the physicochemical analyses of the grapevines’ rhizosphere, soil samples were collected in July 2022. Three randomized sampling zones were established in each vineyard. Rhizosphere soil samples (~ 1 kg) were collected from each zone using an auger at a depth of 20–30 cm, 10 cm wide from the trunk base of the grapevine. Each sampling point was composed of two consecutive subsamples (using two adjacent grapevines), which were collected and mixed to form one composite sample. A total of 12 composite samples were obtained for physicochemical analysis. Composite soil samples were sifted, and small rocks and roots were separated. Afterwards, soil samples were stored at − 20 °C until shipment to A2 Análises Químicas, Lda. (Guimarães, Portugal), where physicochemical analyses were performed using the Mehlich-3 extraction method [14]. For all sampling zones, data were obtained on pH (H₂O), organic matter content (OM), carbon-to-nitrogen ratio (C:N), and macro- and micronutrient contents, including nitrogen (N), phosphorus (P), potassium (K), calcium (Ca), magnesium (Mg), sulphur (S), iron (Fe), manganese (Mn), and copper (Cu). The results were compared with the reference values established for the Mehlich-3 method [14].

### Soil Sampling for Bacterial DNA Metabarcoding

To assess the bacterial rhizosphere microbiome and its dynamics, six randomized rhizosphere soil samples per vineyard (*n* = 6) were collected from the rhizosphere (20–30 cm depth, ~ 10 cm from the trunk base) in July 2022, January 2023, July 2023, and January 2024. Samples from the first time point, used to establish the baseline bacterial community, were collected simultaneously with those for physicochemical analysis; half were co-located with those sites, and the remainder were randomly selected across each vineyard. The nearest grapevines were physically identified at the first time point so that subsequent sampling was performed at the same locations.

DNA extraction was performed using the E.Z.N.A.® Soil DNA Kit (Omega Bio-tek, Inc., GA, USA). DNA integrity was verified by 1% agarose gel electrophoresis, and concentration was measured using an LVis Plate and a FLUOstar® Omega microplate reader (BMG Labtech, Germany). For each vineyard and sampling time point, the six samples were pooled to generate a representative composite sample. High-molecular-weight DNA aliquots with 260/280 and 260/230 ratios between 1.8 and 2.0 were submitted to Novogene Corporation Inc. (Cambridge, UK) for library preparation and sequencing. Despite the pooled samples, which is a limiting factor considering statistical analysis, these pooled samples are representative of each vineyard and time point, reflecting the cumulative relative abundance of each parcel, without intra-vineyard variance.

The V3–V4 hypervariable region of the bacterial 16S rRNA gene was amplified using the primer pair 341 F (CCTAYGGGRBGCASCAG) and 806R (GGACTACNNGGGTATCTAAT) (Novogene Corporation Inc., Cambridge, UK). PCR was carried out in 15 μL reactions containing Phusion® High-Fidelity PCR Master Mix (New England Biolabs, USA), 0.2 μM of each primer, and 10 ng of template DNA. The amplification program consisted of an initial denaturation step at 98 °C for 1 min, followed by 30 cycles of 98 °C for 10 s, 50 °C for 30 s, and 72 °C for 30 s, and a final extension at 72 °C for 5 min. Amplicons of the correct size were selected by electrophoresis on 2% agarose gel, pooled, end-repaired, A-tailed, and ligated to Illumina adapters. Sequencing was performed on an Illumina NovaSeq 6000 platform using paired-end 2 × 250 bp chemistry. Library quality control included quantification by Qubit and real-time PCR, and assessment of fragment size distribution using a bioanalyzer. Libraries were subsequently pooled and purified with the Universal DNA Purification Kit (TianGen, China).

### Microbial Community bioinformatics

#### Data Cleaning and Manipulation

Raw paired-end reads were demultiplexed and assigned to samples based on unique barcode sequences, and barcode and primer sequences were subsequently removed. Sequence processing was performed in QIIME 2 v2024.10 [15, 16] using the DADA2 denoise-paired plugin, which was used for quality filtering, denoising, read merging, chimera removal, and ASV inference [17]. Forward and reverse reads were truncated to 225 and 222 bp, respectively, and filtered using maximum expected error thresholds of 2 and a truncation quality score of 2. Read merging was performed with max-merge-mismatch = 0, denoising used pooling-method = "independent", and chimera removal used chimera-method = "consensus". Taxonomic assignment of the ASVs was conducted using the *feature-classifier* plugin [18] and the SILVA 138.1 ribosomal RNA gene database [19].

#### Core Microbiome, Alpha and Beta Diversity Assessment

To define the baseline composition of the most abundant core microbiome, ASV counts were subsetted to the relevant baseline samples from the four vineyard sites. Bacterial genera were operationalized at the genus level, and counts were converted to relative abundances (%). To create a clear visualization, the top 20 bacterial genera were retained, while lower-abundance genera (less than 1%) were aggregated, as well as the unassigned ones. The relationship between the baseline soil physicochemical properties and the overall structure of the baseline bacterial communities was assessed through an environmental vector fitting.

Soil physicochemical parameters were fitted onto a Bray–Curtis Principal Coordinates Analysis (PCoA) using the *envfit* function from the *vegan* [20] package of RStudio (2026.01.1 Build 403). Subsequently, the influence of soil physicochemical properties on specific bacterial abundances was assessed by aggregating data across the four baseline samples to evaluate ecosystem-wide ecological patterns. Pairwise Pearson correlation coefficients (*ρ*) were computed between the top bacterial genera and the soil physicochemical parameters. Statistical significance of the calculated correlation coefficients was determined, with influences marked as significant when the pairwise p-value < 0.05. Data manipulation, correlation calculations, and generating the final aggregated correlation heatmap were performed in RStudio using the *dplyr*, *reshape2*, and *ggplot2* packages [21–23].

Baseline alpha diversity core metrics were calculated using the *vegan* package in RStudio. To address the uneven sequencing depths across samples, the ASV counts were rarefied to an even depth to the lowest number of reads obtained in a single sample (25.000 reads) using the *rrarefy* function in the *vegan* package (rarefaction curve demonstrated in Supplementary Fig. S1). *α*-diversity descriptive statistics of the baseline time point were then assessed by calculating Observed Features, Shannon’s diversity index (H), and Faith’s Phylogenetic Diversity.

To assess the alpha diversity differences across the entire temporal study, according to vineyard site, rootstock, and seasonality, ASVs abundance matrices from all samples were aggregated. Community richness and overall diversity were assessed for all samples, separated by the focused treatment (vineyard site, rootstock, or seasonality), using the *vegan* package in RStudio. Statistically significant differences were assessed by employing non-parametric tests: the Kruskal–Wallis rank-sum was used to compare variances among the vineyard sites, whilst for binary variables (rootstock and seasonality) the Wilcoxon rank-sum test was used.

To assess the influence of vineyard site, rootstock, and seasonality on the bacterial communities, *β*-diversity encompassing the four time-points was evaluated using PCoA plots based on the Bray–Curtis dissimilarity index, calculated using *tidyverse* and *vegan* [24] packages on RStudio. PERMANOVA (Permutational Multivariate Analysis of Variance,999 permutations) was employed for statistical testing of *β*-diversity.

#### Microbiome Structural Function and Metabolic Pathways Assessment

The evaluation of the microbial communities’ function and the influence of the vineyard site, rootstock, and seasonality on it was assessed by the grouping of bacterial taxa into r-Strategists (copiotrophs) and K-Strategists (oligotrophs) categories, according to [25] and [26]. This categorization was performed by dividing specific bacterial phyla into r-Strategists (Proteobacteria, Bacteroidetes, and Firmicutes) and K-Strategists (Acidobacteria, Actinobacteria, Verrucomicrobia, Planctomycetes, Chloroflexi, and Firmicutes) – Firmicutes is described as having both copiotrophic and oligotrophic bacteria. The relative abundances of individual phyla belonging to each respective strategy were summed per sample using the *tidyverse* package on RStudio. Non-parametric tests were conducted using the native *stats* package in RStudio [27], namely Kruskal–Wallis rank-sum to compare variances among the vineyard sites, and the Wilcoxon rank-sum test for binary variables (rootstock and seasonality). For data visualization, the mean relative abundance and standard error (SE) were calculated for each vineyard and strategy combination.

Also, a macro-metabolic profile between rootstocks was performed to identify the mean expressed functional genes, and the number of significantly enriched functions, grouped by primary KEGG Ortholog (KO) categories [28, 29]. This community function was predicted from the 16S rRNA gene data, using PICRUSt2 (Phylogenetic Investigation of Communities by Reconstruction of Unobserved States) [30], which allowed the inference of the genomic functional potential of the rhizosphere microbiome by aligning ASVs to a reference phylogenetic tree and predicting the abundance of KOs. A meta-analysis was performed using *tidyverse* in RStudio, and predicted functions with significantly different means and abundances between rootstocks (Wilcoxon rank-sum test, *p* < 0.05) were mapped into macro-metabolic categories. To ensure robustness and prevent false positives, a stringent prevalence filter before the Wilcoxon rank-sum tests was implemented, retaining features with a presence minimum in 25% of the samples [31]. This categorization followed the KEGG pathway hierarchy, grouping individual genes into core functional blocks, calculating the total number of unique, significantly enriched pathways within each macro-category of each rootstock.

## Results

### Site Characterization and Baseline Physicochemical Characterization

#### Site Characterization and Terroir Properties

Terroir characterization associates environmental, genetic, and cultural characteristics that influence the grapevine in its production.

The four vineyards share certain characteristics, such as the grapevine variety used (Tinta–Roriz) and the production system, which is integrated in all cases. However, several parameters differ across the sites, indicating distinct terroirs. These include variations in altitude, landscape, rootstock selection, vineyard age, and soil fertilization (Table 1).

#### Physicochemical Properties

The physicochemical analysis performed in July 2022 provided a robust characterization of vineyard terroir and revealed significant differences among sites (Table 2). EM and BL differed significantly in soil pH: BL was at the lower end of the reference range, whereas EM exceeded the recommended values, indicating alkaline soil conditions. For the C:N ratio, only SX fell outside the reference range, suggesting low carbon content. Total nitrogen was deficient in all vineyards (< 0.07%; Supplementary Table S1), and organic matter content was also below the reference values at all sites (Table 3). Phosphorus, Mn, and Cu were within the optimal range across all vineyards; however, Mn differed significantly between SX and EM, and Cu differed significantly among all vineyards (Table 3). Potassium was the most unbalanced nutrient, with excessive levels in CV and deficient levels in the remaining vineyards. Calcium exceeded the recommended values in all vineyards except CV, where it remained within an adequate range. Magnesium was extremely high in SX, BL, and EM relative to the reference values, whereas CV remained within the adequate range (Table 2). Fe availability in the soils of CV, SX, and BL was within the reference range, whereas EM showed concentrations above the optimal values. In contrast, only SX presented an adequate sulphur (S) concentration, while the remaining vineyards showed S deficiency, with BL and EM falling below the recommended threshold (Table 2).

**Table 2:** Soil physicochemical properties of the four sampled vineyard sites (CV, Cavernelho; SX, Seixo; BL, Belém; EM, Ervamoira) (n = 3) and reference values expected for this region and crop

Trait	**CV**		**SX**	**BL**	**EM**	**Reference **^**1**^ [14]
pH (H_2_O)	6.77 ± 0.40ab^2^	6.83 ± 0.31ab		6.03 ± 0.35b	**7.23 ± 0.06a**	6.0—7.0
C:N Ratio	11.67 ± 1.15a	**9.33 ± 0.31b**		10.30 ± 2.14ab	10.00 ± 0.95ab	10.0—12.0
Organic matter (%)	**1.23 ± 0.35a**	1*		**1.01 ± 0.02a**	**1.10 ± 0.16a**	2.0—4.0
P (mg/kg P_2_O_5_)	93.75 ± 8.41a	89.55 ± 31.75a		74.97 ± 27.51a	94.53 ± 10.35a	98—162
K (mg/kg K_2_O)	**404.50 ± 53.03a**	**41.93 ± 12.64bc**		**48.00 ± 6.35b**	**33.60 ± 2.23c**	74—140
Ca (mg/kg CaO)	1298.67 ± 465.51b	**2890.00 ± 133.43a**		**1494.33 ± 414.52b**	**3596.67 ± 404.92a**	819—1047
Mg (mg/kg MgO)	174.33 ± 27.47c	**754.33 ± 133.32a**		**377.33 ± 25.72b**	**737.67 ± 78.23a**	100—205
Fe (mg/kg Fe)	76.23 ± 16.38c	120.50 ± 24.56ab		104.57 ± 13.72bc	**148.33 ± 15.04a**	50—100
Mn (mg/kg Mn)	37.00 ± 25.12ab	22.97 ± 9.43b		**17.30 ± 4.46b**	63.63 ± 10.32a	25—100
Cu (mg/kg Cu)	17.45 ± 0.21a	8.75 ± 0.21b		0.50 ± 0.10c	4.30 ± 0.26d	0.5—20
S (mg/kg S)	**8.83 ± 0.57a**	10.3 ± 1.71a		8.5*	8.5*	> 10

^1^ Reference values adopted accordingly to the Mehlich-3 quantification method; Bold values indicate values out of the reference; ^2^ For each trait (row), sites with different letters differ significantly; * Indicates results below the threshold of the test

**Table 3:** Alpha diversity baseline metrics (Summer 2022) of the four vineyards in the study

Vineyard	Observed Features	Shannon (H')	Faith’s PD
CV	273	2.56	137.50
SX	254	2.41	123.40
BL	292	2.61	144.20
EM	283	2.61	124.50

### Bacterial Community Baseline Structure and Diversity

#### Community Structure Baseline, Alpha and Beta Diversity

**Fig. 2:**
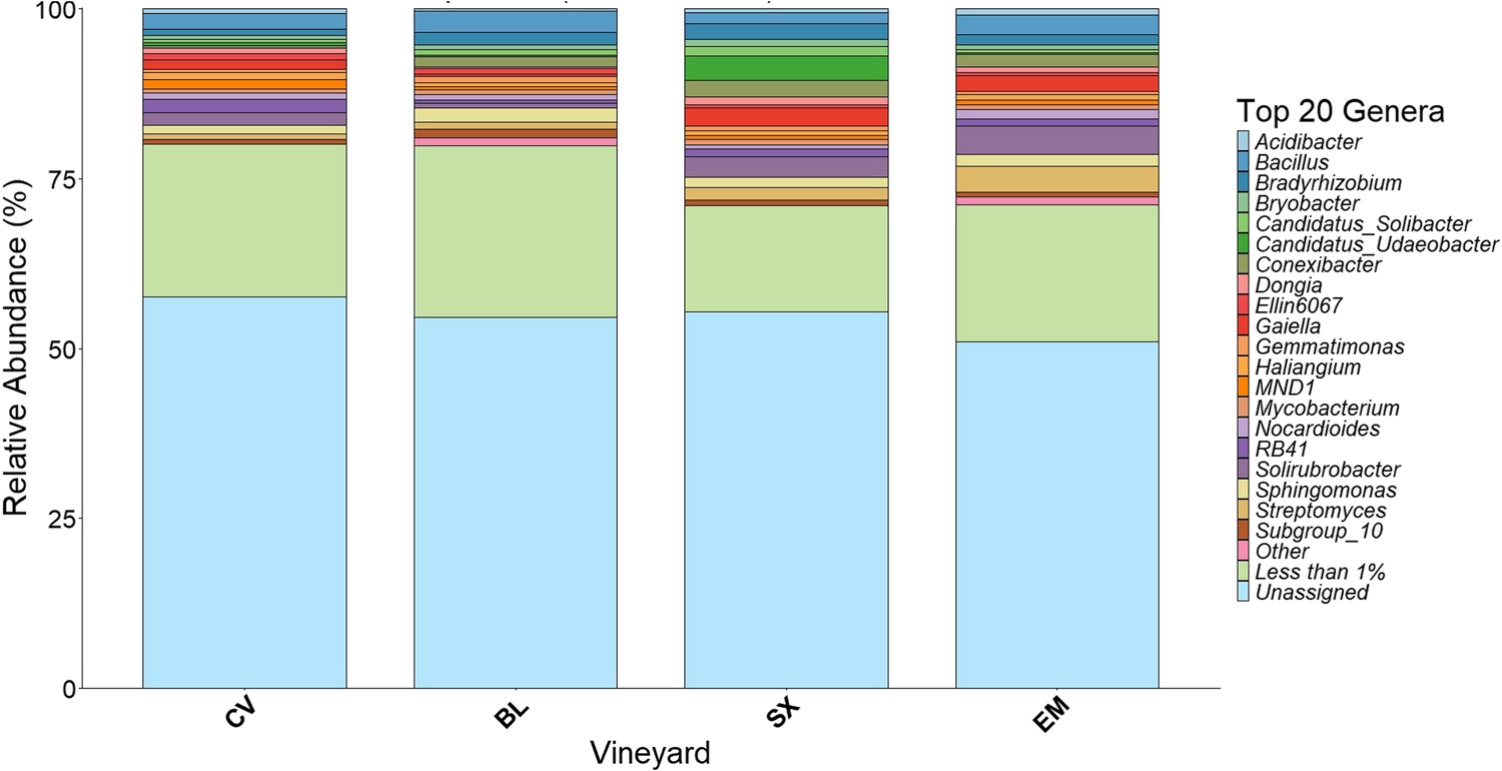
Relative abundance of the top 20 most abundant bacterial genera in rhizosphere soil samples in the baseline data (Summer 2022). Each color represents a different genus. “Less than 1%” includes all bacteria with less than 1% of relative abundance. “Other” includes the combined relative abundance of all genera outside the top 10 for each sample. Unassigned refers to ASVs that could not be taxonomically classified or were not resolved to the genus level

The analysis of the rhizosphere core bacteriome, through the quantification of relative abundances of the Top 20 genera, revealed that *Bacillus*, *Solirubrobacter*, *Streptomyces*, *Sphingomonas* and *Bradyrhizobium* (average percentages, across the four vineyards, of 2.46%, 2.43%, 1.93%, 1.62%, and 1.69%, respectively) seem to be the most dominant genera of the bacterial microbiome composition in the baseline time point, Summer 2022 (Fig. 2). The “less than 1%” group had higher relative abundance in BL, and lower relative abundance in SX, suggesting a more diverse community in BL than in SX (See complementary data – 2). Despite the differences in the soil’s physicochemical properties mentioned earlier, the four vineyards shared a similar core rhizosphere bacterial structure, as most of the cited genera in the relative abundance analysis are present in the four respective communities (Supplementary Table S2).

The baseline assessment of the rhizosphere bacteriome established the initial ecological profile of the four vineyards. The representative pooled samples suggest different gradients in bacterial richness and diversity. BL and EM samples, having higher observed features (292 and 283, respectively) and Shannon indices (H’ = 2.61), indicate that the communities composing these vineyards are the richest of the four, and have the most balanced/evenly distributed communities. However, Faith’s PD indicates that the most evolutionarily different bacterial communities belong to BL, followed by CV. These results suggest that these vineyards have more favourable conditions for a wider bacterial community to prosper. Even though EM’s rhizosphere bacteriome had higher richness and diversity, this community is composed of more evolutionarily close individuals. Conversely, the poorer initial community appears to be in SX, having the lowest scores in all alpha diversity metrics, suggesting a less prosperous environment for bacterial proliferation.

#### Soil Profiling and Influence On the Baseline Microbiome

**Fig. 3:**
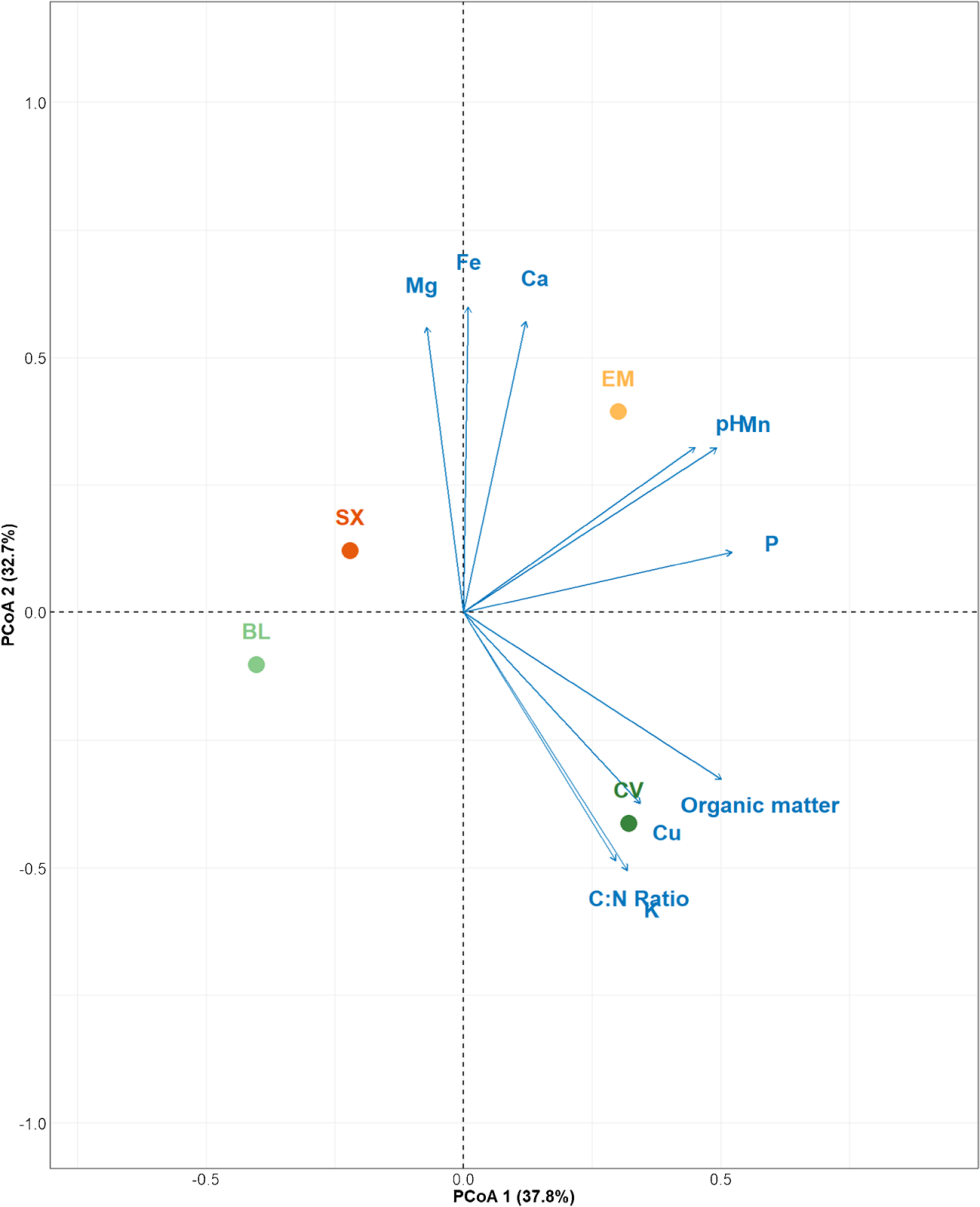
Principal Coordinates Analysis (PCoA) based on a Bray–Curtis dissimilarity matrix of the baseline rhizosphere microbiome configuration in Summer 2022, linked to soil physicochemical properties vectors

The relationships between the soil physicochemical properties and the overall structure of the bacterial communities were investigated through an environmental vector fitting on a Bray–Curtis Principal Coordinates Analysis (PCoA, Fig. 3). The PCoA show a substantial proportion of the dissimilarity attributed to the first two axes (PCoA 1 explains 37.8% of the total dissimilarity, whilst PCoA 2 explains 32.7%), and a clear spatial differentiation of the four vineyards, which suggests different bacterial communities among them. However, due to the lack of replicates, a PERMANOVA to assess the significance of these differences was not possible. PCoA 1 seems to describe the nutritional richness of the vineyards, suggesting that vineyards on the right quadrants have richer soils, whilst the ones on the left side are poorer. PCoA 2 suggests differentiation between soil profiles, as the upper–quadrants vineyards have more mineral soil profiles, whilst the ones on the downside appear to have more organically-enriched soils. Overall, the results indicate that CV is characterized by higher organic matter, Cu and K contents, and a higher C:N ratio. EM was positively associated with elevated concentrations of several elements, including Fe, Ca, Mn, and P, as well as with a more basic pH. In contrast, SX and BL exhibited weaker associations with most physicochemical traits. BL was negatively associated with pH and P concentration, whereas SX was negatively associated with organic matter and C:N ratio, while showing a positive association with Mg.

#### Influence of Soil Profile On Specific Bacteria

**Fig. 4:**
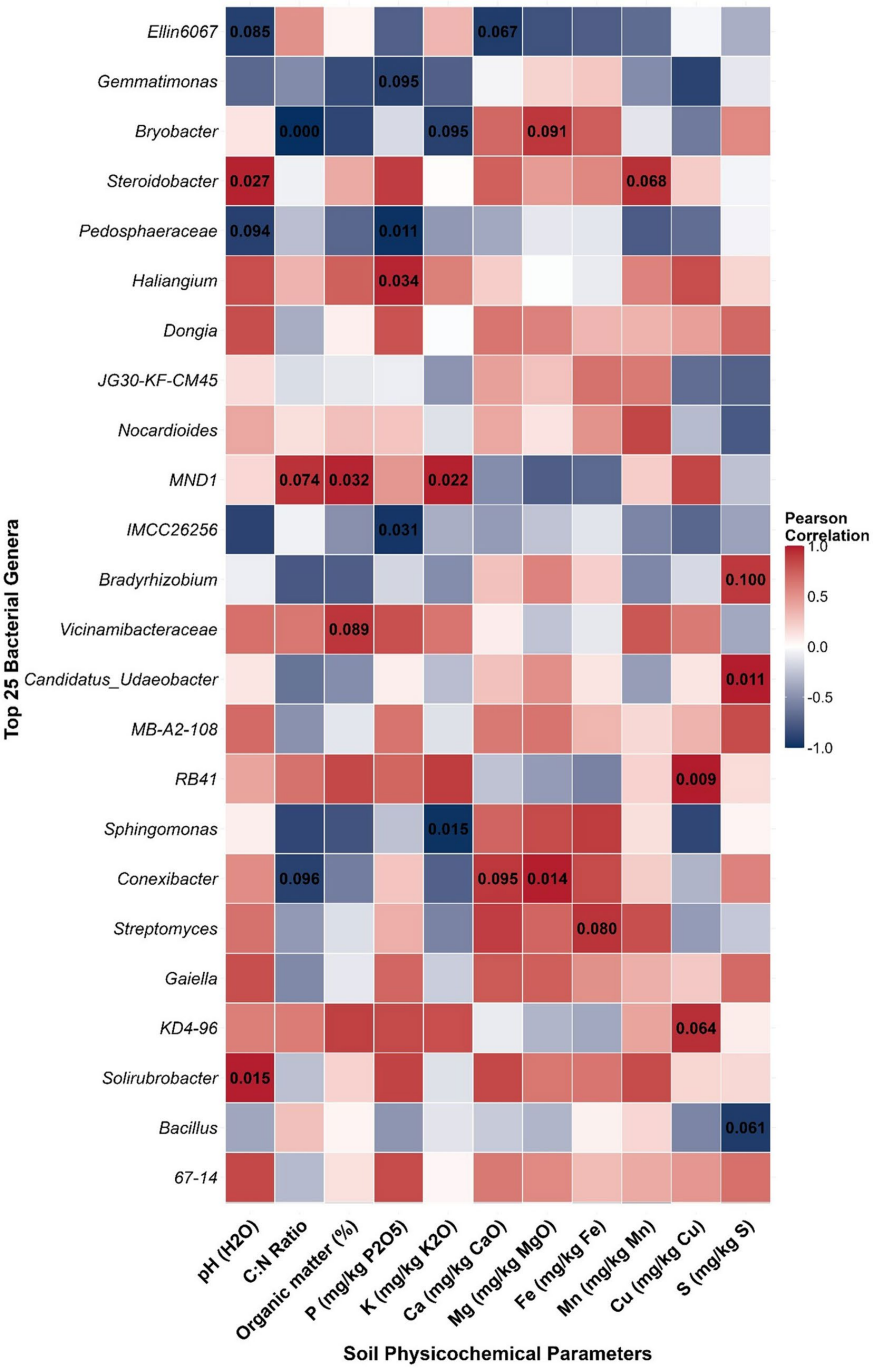
Heatmap illustrating aggregated Pearson correlation patterns between soil physicochemical variables and the relative abundance of the 25 dominant bacterial taxa in baseline vineyard soils. Red indicates positive correlations, whereas blue indicates negative correlations. Correlations with p < 0.05 were considered statistically significant

Results indicate several positive correlations between bacterial genera relative abundance with specific nutrients and soil properties (Fig. 4), such as *Steroidobacter* and *Solirubrobacter* with pH, of *MND1* with organic matter and potassium, of *Haliangium* with phosphorous, of *Conexibacter* with magnesium, of *RB41* with copper, and of *Candidatus*_*udaeobacter* with sulfur.

Negative correlations were also observed, such as that of *Bryobacter* with the C:N ratio, of *Pedosphaeraceae* and *IMCC26256* with phosphorus*,* and of *Sphingomonas* with potassium. Despite the absence of significant results, some genera appear highly correlated with certain parameters, such as *Streptomyces* with Fe, *Bradyrhizobium* with sulfur, and *Steroidobacter* with manganese.

These results suggest a direct influence of the soil profile on the baseline bacterial microbiome, namely by shaping overall structures and influencing the abundance of specific genera.

### Influence of Terroir, Genetic, and Seasonal Variables On the Rhizosphere Bacterial Community

To explore the complex dynamics of the bacterial communities of the rhizosphere, and at what level they are influenced by genetic and seasonal variables, a two-year longitudinal comprehension of the influence of the vineyard's context, and the specific evaluation of the impact of the rootstocks and seasonality on the modulation and selection of the rhizosphere microbiome will be performed.

#### Influence of Vineyard, Rootstock, and Season On Alpha Diversity

**Fig. 5:**
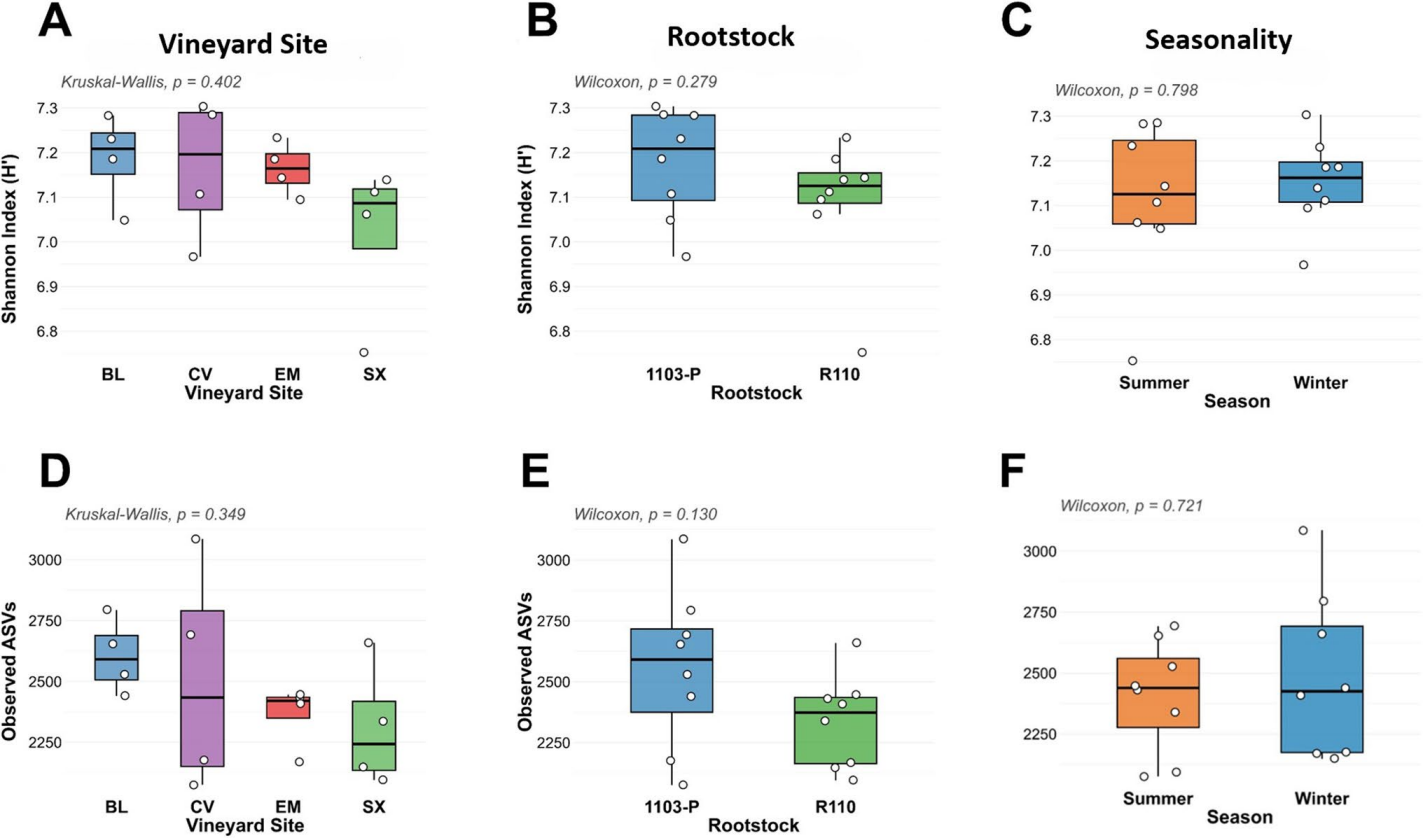
Alpha diversity analysis of the influence of the vineyard site, rootstock, and seasonality on the two-year sampled rhizosphere bacterial communities: A, B, C – Shannon Diversity Index (H´); D, E, F – Number of Observed ASVs; white dots represent each replicate included in the statistical analysis

Firstly, alpha diversity metrics were explored, and results are presented in the boxplots of Fig. 5. No significant differences in bacterial richness or evenness were observed among vineyard sites, as indicated by the Shannon index (*p* = 0.402) and observed ASVs (*p* = 0.349). Similarly, rootstock had no significant effect on alpha diversity (Shannon index, *p* = 0.279; observed ASVs, *p* = 0.130). Seasonality also did not significantly affect the richness or evenness of rhizosphere bacterial communities in the vineyards (Shannon index, *p* = 0.798; observed ASVs, *p* = 0.721).

#### Influence of Vineyard, Rootstock, and Season On Beta Diversity

**Fig. 6:**
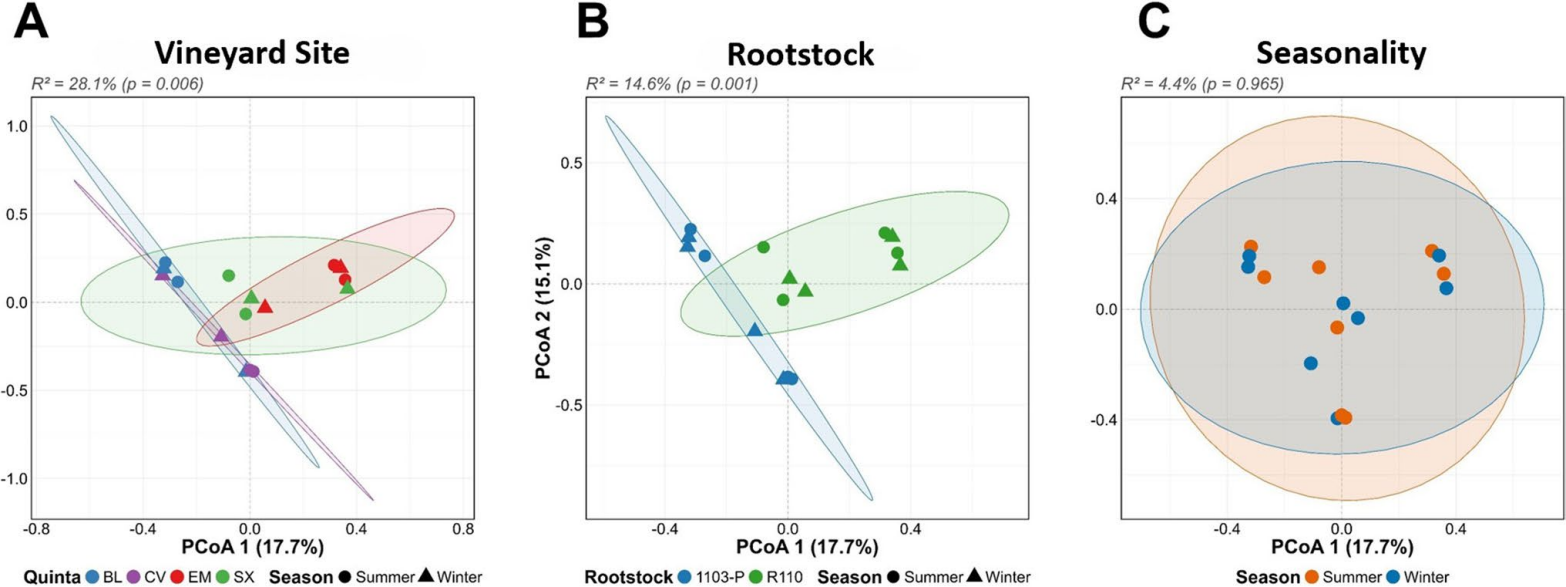
Principal Coordinates Analysis (PCoA) ordination based on a Bray–Curtis dissimilarity matrix illustrating the influence of vineyard site (A), rootstock (B), and seasonality (C) on community structure across samples collected over the two-year sampling period. Group differences were assessed by PERMANOVA

The results of the Bray–Curtis dissimilarity matrix, supported by PERMANOVA (Fig. 6), show distinct microbiome structures between vineyards, with 28.1% of the total variability within these communities attributable to overall *terroir* (*p-value* = 0.006). Results suggest that these differences are, in part, explained by the rootstock variable, as 14.6% of the total variability is attributed to the genetic factor (*p-value* = 0.001). Furthermore, seasonal variability did not have a significant influence on the microbiome structures in our context, as only 4.4% of the total variability is attributed to seasonality (*p-value* = 0.965), meaning that 9.1% of the variability could be explained by the remaining field variables.

These results must be understood under the full context of the vineyards, as they are obviously inserted in field conditions with multiple environmental and cultural variables, which do not allow definitive conclusions in some cases, as they are confounding factors.

### Impact of Terroir, Genetic, and Seasonal Variables On the Microbiome’s Functional Pathways

#### Copiotrophic vs. Oligotrophic Bacteria: The Variability of the Bacteriome Functionality

**Fig. 7:**
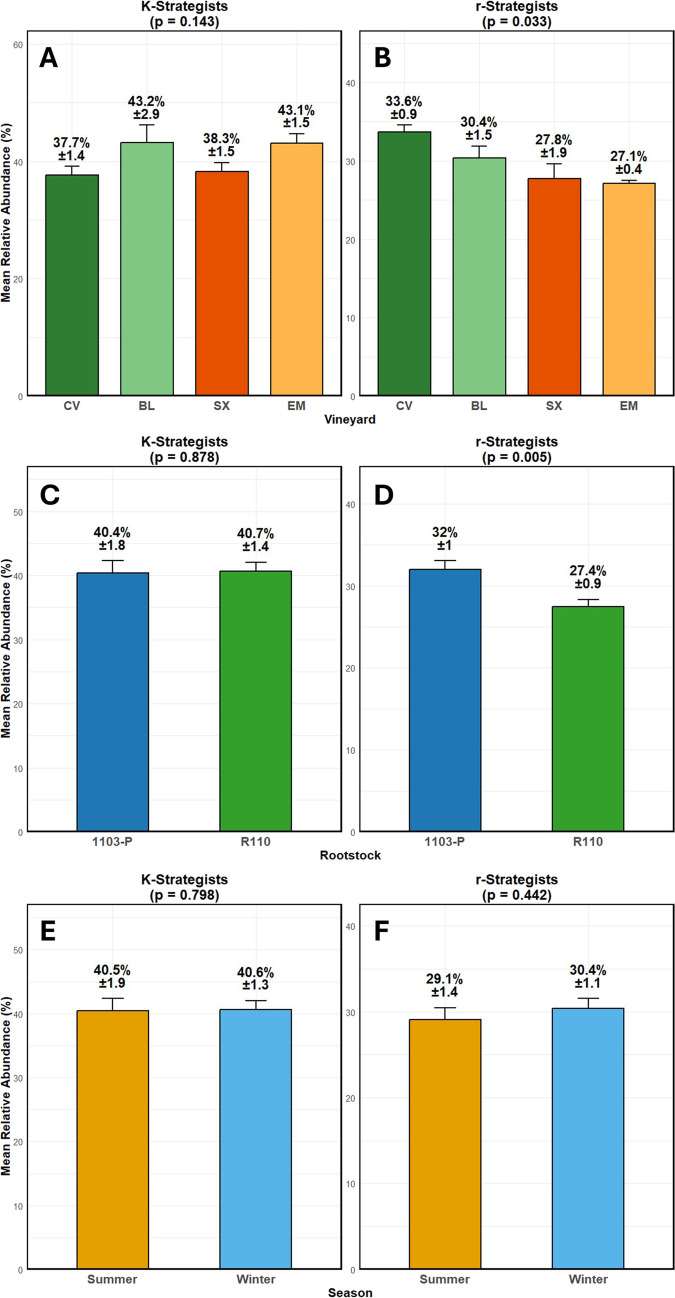
Mean relative abundance (%) of the K-Strategists and r-Strategists groups, and their respective standard deviations, of the average values of the two-year sampling period; A, B – Vineyard Site; C, D – Rootstock; E, F – Season; p-values < 0.05 mean statistically significant results

To assess the influence of the vineyard, rootstock and seasonality variables on the functional characteristics of the bacterial communities, a phylum differentiation between “r-Strategists”, referring to copiotrophs, and “K-Strategists”, referring to oligotrophs, was performed in the communities under analysis (Fig. 7).

Significant results were observed in the “Vineyard” variable regarding r-Strategists community (*p-value* = 0.033), influenced by the differences between the communities of CV and EM, and the close to significance results between the ones of CV and SX (Supplementary Table S3). Regarding the K-Strategists communities between vineyards, no significant differences were observed, despite the near-significantly different results between CV and EM (*p-value* = 0.057).

Concerning the results of the “Rootstock” variable, functionally different communities were observed, as the 1103-P rootstock promoted a higher abundance of r-Strategists (*p-value* = 0.005). However, regarding K-Strategists, no significant differences were observed, suggesting that the near-significantly different results seen in the “Vineyard” variable were not due to the influence of rootstocks.

Seasonality did not influence either r-Strategists or K-Strategists, suggesting that it has no impact on these functional groups’ composition. Furthermore, neither rootstocks nor seasonality explained the observed tendency in the K-Strategists relative abundance in the “Vineyard” variable, suggesting that some other variable not analyzed in our work might have some influence on this functional group.

Overall, these results indicate that there are differences in the functional pathways and environment-adaptation characteristics of the analyzed microbiomes, and that these differences are, at least in part, attributed to the rootstock type.

#### The Influence of Rootstocks On Functional Diversity and Intensity

**Fig. 8:**
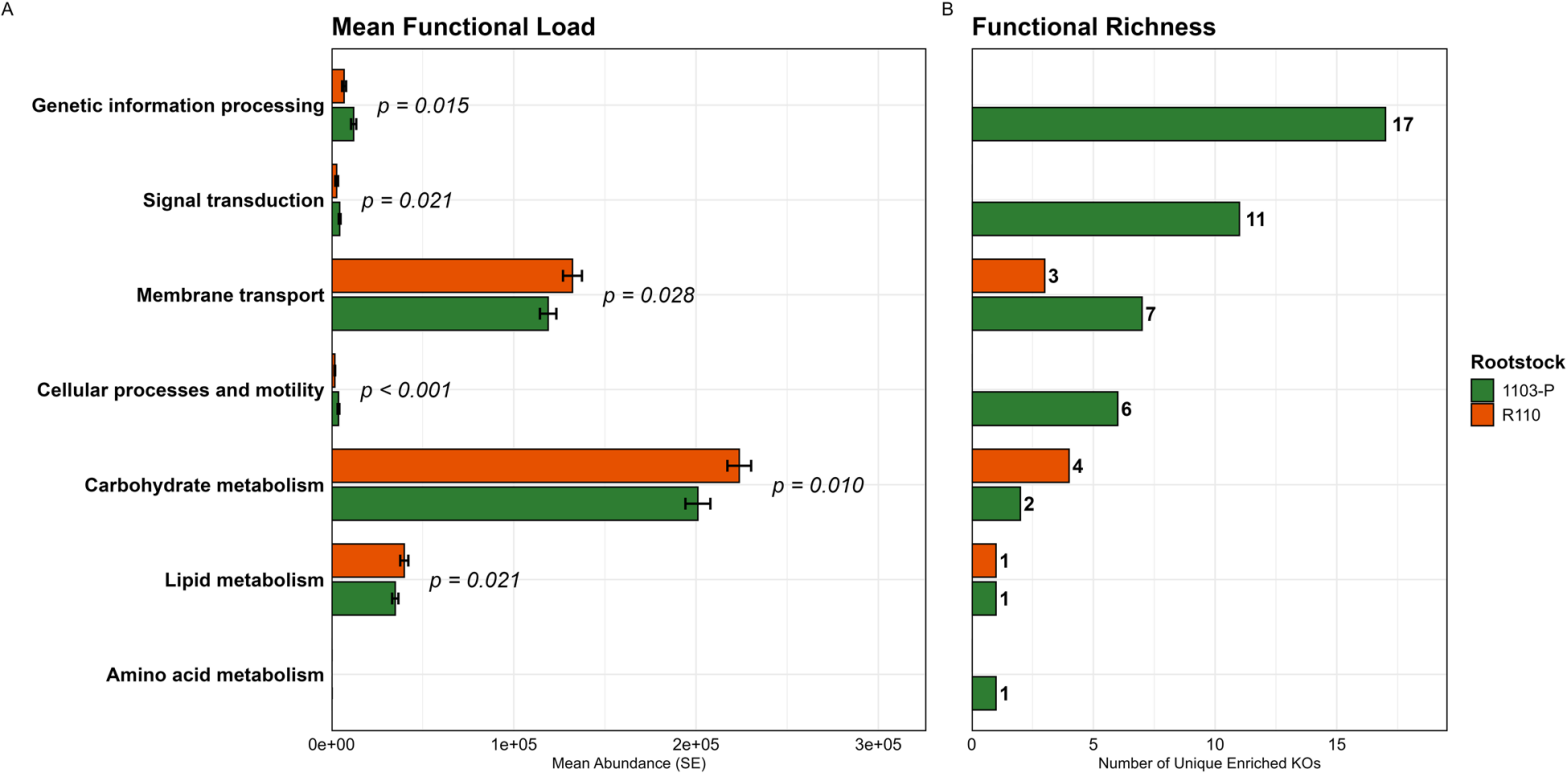
Mean predicted functional gene expression of the two-year samples, and respective Wilcoxon rank-sum tests (A); Aggregated count of predicted statistically significant enriched functions (p-value < 0.05, prevalence > 25%) determined by KOs associated with each metabolic pathway (B); Community function was predicted from the 16S rRNA gene data, using PICRUSt2

Regarding the differences observed in functional pathways and environment-adaptation characteristics attributed to rootstocks and vineyards, the assessment of how they alter the metabolic pathways must be conducted. Therefore, a differential functional diversity assessment was performed, namely by quantifying the mean predicted functional gene expression, and the diversity of genes significantly influenced by each rootstock, and by placing them into their respective metabolic pathways (Fig. 8).

Results suggest distinct influences of both rootstocks on the functional gene expression of their respective associated bacterial communities. The 1103-P rootstock had significantly higher means for predicted functional gene expression in “Genetic information processing”, “Signal transduction”, and “Cellular processes and motility”, whilst the R110 rootstock had significantly higher means in “Membrane transport”, “Carbohydrate metabolism”, and “Lipid metabolism” (Fig. 8A). These results suggest different behaviours and strategies of the bacteria surrounding the rootstocks.

Furthermore, results also suggest that the differences between the communities’ functional pathways had more unique enriched gene expression associated with “Genetic information processing” and “Signal transduction” mechanisms, which were higher for the communities associated with the 1103-P (Fig. 8B). “Membrane transport”, “Cellular processes and motility”, and “Amino acid metabolism” have also had more predicted aggregated counts of enriched functions in the 1103-P. Contrarily, “Carbohydrate metabolism” had more aggregated counts related to the R110 rootstock. Overall, a general increase in unique gene expression in the communities on the rhizosphere of the 1103-P rootstock was predicted, suggesting a more diversified transcriptome in the bacterial communities associated with it.

## Discussion

The microbiome is a functional extension of the grapevine’s genome, and has been proven to be fundamental regarding plants’ activity, as it plays several roles in supporting plant nutrition, increasing the grapevine’s tolerance to stress, and controlling pathogen proliferation [32–34]. However, several environmental and cultural factors modify the microbial communities, thereby shaping their structure and ecological roles [9, 35–38].

Our results suggest a multi-factor influence on the rhizosphere bacterial communities in the DDR, where the physicochemical characteristics of the soil act as an environmental filter to the bacterial community, whilst the rootstock genotype exerts a selective influence, namely by influencing the metabolic functionality.

### Baseline Communities and Soil Physiochemistry As an Environmental Filter

The baseline ecological profiling of the vineyards showed that the bacterial populations of the rhizosphere were composed of distinct communities, mainly differing in richness and phylogenetic diversity. These communities were primarily shaped by physicochemical properties, with mineral nutrient content, organic composition, and pH emerging as the main factors driving their differentiation.

It is well documented that organic matter promotes alpha diversity, as it is a complex “cocktail” of different carbon sources, creating a vast number of ecological niches and thereby establishing the conditions for a wide variety of bacteria to coexist [39, 40]. Furthermore, due to the particle-aggregation capacity of organic matter, it develops a complex network of micro-pores, which creates a heterogeneous micro-environment, allowing for a higher number of different bacteria to share the same physical space, and increasing soil water retention capacity [39, 41]. CV’s alpha diversity metrics and the PCoA accurately suggest a strong relationship between a broader bacterial community and higher organic matter and carbon content.

Contrarily, inorganic soils are often associated with lower-diversified bacterial communities, as they are intrinsically poorer in organic matter and carbon sources. These conditions deplete the copiotrophic bacteria’s chances of survival, whilst the oligotrophic ones, with slower metabolisms, are better adapted [25, 26, 42].

Also, a mineral soil is physically and chemically more homogeneous, which stimulates competition between bacteria and decreases the capacity to retain water, leading to lower diversity and richness of the community [43]. SX alpha diversity metrics seem to fit this scenario, as it correlates with a more inorganic soil with low carbon content. In fact, SX low C:N ratio could cause a significant “carbon hunger” and, combined with the lack of water in the soil, modulate the bacterial community toward a more functionally specialised role in mobilising inorganic resources [42],Cruz-Silva et al. 2023).

Furthermore, SX has a harsher environment, as it is established in a scarp, which favours water runoff, further decreasing the chances of water retention in soil [44–46]. This topographic characteristic may contribute to the increased differences found in the baseline microbiome genetic structure among these *terroirs*, as lower water availability decreases bacterial taxonomic/functional diversity. This reinforces the hypothesis that physicochemical properties and landscape are key environmental modulators of these communities, although landscape was not directly evaluated in this work [6, 47–49].

Soil pH seems to have some influence on the modulation of the baseline microbial communities, especially in BL (negative correlation) and EM (positive correlation). In fact, pH is a fundamental chemical parameter, as it manipulates nutrient solubility in the soil, modifying their availability for plants and bacteria to use. The soil pH has an ideal value range for bacterial richness and diversity between 6.0–7.0 [50], and is reported to be negatively correlated with microbial communities, decreasing carbon storage and, therefore, lowering the organic matter content [3, 51, 52]. However, some bacteria are favoured by the increasing pH, such as shown by our results of *Solirubrobacter* and *Steroidobacter* in the correlation matrix, proving that it is an ecological filter that changes the bacterial community’s structure [53–55]. Indeed, pH values outside the ideal range restrict nutrient availability due to precipitation into insoluble forms [56], promoting communities better adapted to limited-resource environments, and efficient strategists in oligotrophic soils, such as *Solirubrobacter* [26, 57–59]. Considering this, it would be expected that the neutral pH seen in BL provides optimal conditions for a broad, phylogenetically diverse community to proliferate, whilst on EM, it forces the community to a more specialized and phylogenetically clustered one, reflected by the lower Faith’s PD observed in this vineyard.

However, it is noticeable that a resilient core microbiome dominates the four locations, which could reflect a distinctive, strong microbial signature of the Douro Demarcated Region, although this thesis should be further explored.

### The Influence of Rootstock and the Environment On the Microbiome’s Structural Function

The microbial community present in the bulk soil is considered a “seed bank” to which the plants recur to assemble the plant-associated microbiome [60]. Our findings demonstrate that the rhizosphere microbiome is modulated by a multi-factor interaction, comprising environmental filters that compose the *terroir* and that could be a primary modulator, and by a functional modulation by the rootstock, which could be exerting a secondary selection. Overall, the *terroir* multi-factors explain 28.1% of the variability, 14.6% of those being related to the rootstock influence.

One of the main environmental filters influencing the rhizosphere microbiome is the soil physicochemical properties, which we have seen exert selective pressure on the structure of the microbiomes of the baseline. Nevertheless, despite the soil physicochemical properties being modified at a slow pace, we assumed that it is part of the *terroir* in our analysis, due to the lack of temporal replicates in our experimental design to properly analyze its individual effect during the two-year sampling. Variables such as climate, landscape, and cultural practices (agrochemical use, tillage, fertilizer applications) also compose the *terroir*, and are reported to influence the microbial communities of the soil, although not quantifiable in our work [44, 46–49, 61–63].

The “rootstock” should be considered another variable comprising the *terroir*. Operating in the available “seed bank”, the rootstock genotype is hypothesized to exert a secondary functional selection through the release of differentiated, tailored exudates that attract specific bacteria among the available, thereby developing a metabolic synchronization with the host plant [1, 8, 60, 64]. This metabolic synchronization maximizes plant–microbe interaction, creating a mutualistic relationship, which grants the microbiome access to energy sources, whilst facilitating the uptake of nutrients and improving the usage of the resources stored in the soil, by the plant [6, 54, 60, 65–68].

The comparative analysis indicates that the 1103-P is recruiting a functionally versatile bacterial community, enriched with copiotrophic individuals, belonging to the Proteobacteria and Bacteroidetes phyla. This is likely driven by 1103-P capacity to prioritize exudate quantity and composition regardless of the climate, supported by a positive regulation of aquaporins, which promotes a strategy to maintain the water flux and the physiological activity of the grapevine for longer periods [7, 12, 13, 67, 69, 70]. The maintenance of the exudate flux is fundamental to attract and keep a widely diverse bacterial community, mainly among the r-Strategists group, which is highly dependent on carbon energy sources [71, 72], and highly capable of propagation and growth, metabolic activity, and environmental exploration, as suggested by the functional results in “Genetic information processing”, “Signal transduction” and “Cellular processes and motility”, classic hallmarks of r-Strategists [26, 72].

In contrast, the R110 rootstock has a more conservative approach, tailored to extreme edaphic and climatic conditions. The R110 limits water loss as a priority response to drought stress [12], which acts as a stringent filter to the surrounding microbiome, as it decreases its exudate volume and thereby lowers copiotrophic bacteria diversity on our R110-grafted vineyards. However, despite becoming a less diversified microbiome, our functional results suggest a highly specific metabolic synchronization [60]. The restriction of broad carbon sources diminishes copiotrophic diversity, but selectively enhances a specialized microbiome, with a huge dedication to carbohydrate metabolism and membrane transport, prioritizing and optimizing the uptake of targeted root secretions, relegating expansionist metabolic activities [73–75]. This specific functional architecture is a classic example of a substrate-driven assembly, in which the microbiome is modulated to target specific root secretions in resource-limited environments [76].

Regarding the oligotrophic community, no significant differences were seen between rootstocks, suggesting that these bacteria are less dependent on the radicular exudates. In fact, oligotrophic bacteria have specialized mechanisms to obtain energy from inorganic resources, which are not dependent on the genetic structure of the grapevine [72, 73]. This supports our proposed hierarchical hypothesis, as oligotrophic bacteria are less dependent on root exudation, but instead seem to rely on the physicochemical characteristics of the soil and its inorganic and recalcitrant resources [73, 77].

Overall, the rootstocks’ functional selection was a significant factor influencing the total variability of the microbiome. However, it did not explain all the variability observed in the *terroir* (13.5% was not explained by rootstock). The remaining variability percentage could be attributed to several factors, such as seasonality (which results suggested to be 4.4%, suggesting a high plant-microbiome system resilience), physicochemical properties, landscape, and cultural practices, such as agrochemical use, fertilizer use, or tillage, which are also reported to influence microbial communities [46, 61–63, 78, 79].

While our data align with the proposed hierarchy throughout our work, it is important to acknowledge intrinsic methodological and experimental limitations, common in commercial vineyard field trials. First, to establish a robust representation of the rhizosphere communities of each vineyard, six biological replicates per vineyard were collected and pooled before sequencing. Consequently, the four time-points were the effective unit of replication for the site and rootstock statistical contrasts. Also, multiple variables co-vary across the four sampled sites. Without formal variance partitioning on strictly separable variables, the isolated influence of the rootstock genetics versus the environmental background remains statistically confounded. Therefore, our hypothesis of a hierarchical model, in which the soil physiochemistry acts as a primary environmental filter, and the rootstock exerts a secondary functional selection, should be presented as a robust hypothesis to be formally tested in future better-replicated, strictly controlled experimental designs, rather than as definitive proof.

## Conclusions

This work proposes a hierarchical model for the assembly of the rhizosphere bacterial communities of grapevines in the DDR. Our findings suggest that the physicochemical properties of the soil, mainly organic matter content and pH, which determine the organic/inorganic profile, likely act as a primary environmental filter. This ecological profiling drives the composition of the “seed bank” available to be recruited by the grapevine. Subsequently, the rootstock appears to exert a secondary selective modulation of the bacterial microbiome, filtering the community based on its functional traits. Results suggest that copiotrophic bacteria were modulated by both primary and secondary selective pressures, whilst oligotrophic bacteria were not influenced by rootstock selection, being more dependent on the primary environmental filter. The 1103-P rootstock recruited a more versatile, exploratory microbiome, enriched with copiotrophic bacteria with high motility and growth rate. The R110 selected a highly specialized community, focused on carbohydrate metabolism and membrane transport, optimizing resource use.

In conclusion, this work shows that soil health is fundamental to the “bank seed” diversity, and that the selection of the rootstock is important to attract the most suitable microbiome to a certain condition, as it not only modifies the taxonomy of the community, but reprograms the associated functional structure. These insights are fundamental to the development of sustainable, ecological, and precise viticultural strategies, increasing available knowledge in the soil–plant-microbe interaction to maximize grapevine health and resilience to the upcoming challenges in the DDR.

## Supplementary Information

Below is the link to the electronic supplementary material.

Supplementary file1 (DOCX 49 KB)

## Data Availability

The data that supports the findings of this work will be made public when it is accepted for publishing.

## References

[1] Darriaut R, Lailheugue V, Masneuf-Pomarède I et al (2022) Grapevine rootstock and soil microbiome interactions: keys for a resilient viticulture. Hortic Res 9:019. 10.1093/hr/uhac019 10.1093/hr/uhac019 PMC8985100 35184168

[2] Bokulich NA, Thorngate JH, Richardson PM, Mills DA (2014) Microbial biogeography of wine grapes is conditioned by cultivar, vintage, and climate. Proc Natl Acad Sci U S A 111:E139–E148. 10.1073/pnas.1317377110 24277822 10.1073/pnas.1317377110 PMC3890796

[3] Zarraonaindia I, Owens SM, Weisenhorn P et al (2015) The soil microbiome influences grapevine-associated microbiota. mBio. 10.1128/mBio.02527-14 25805735 10.1128/mBio.02527-14 PMC4453523

[4] Jáuregui I, Ancín M, García-Mina JM et al (2025) Permanent crop cover as a strategy for drought-resistant viticulture: insights on how rhizosphere metagenomics influences leaf-level -omics for an enhanced overall plant response. Front Plant Sci 16:1543171. 10.3389/fpls.2025.1543171 40510172 10.3389/fpls.2025.1543171 PMC12158955

[5] Oro CED, Saorin Puton BM, Venquiaruto LD et al (2024) Effective microbial strategies to remediate contaminated agricultural soils and conserve functions. Agronomy 14(11):2637. 10.3390/agronomy14112637

[6] Lailheugue V, Darriaut R, Tran J et al (2024) Both the scion and rootstock of grafted grapevines influence the rhizosphere and root endophyte microbiomes, but rootstocks have a greater impact. Environ Microbiome 19:24. 10.1186/s40793-024-00566-5 38654392 10.1186/s40793-024-00566-5 PMC11040986

[7] Zuzolo D, Ranauda MA, Maisto M et al (2023) The rootstock shape microbial diversity and functionality in the rhizosphere of *Vitis vinifera* L. cultivar Falanghina. Front Plant Sci 14:1205451. 10.3389/fpls.2023.1205451 37645461 10.3389/fpls.2023.1205451 PMC10461393

[8] Marasco R, Rolli E, Fusi M et al (2018) Grapevine rootstocks shape underground bacterial microbiome and networking but not potential functionality. Microbiome 6(1):3. 10.1186/s40168-017-0391-2 29298729 10.1186/s40168-017-0391-2 PMC5751889

[9] O’brien F, Nesbitt A, Sykes R, Kemp B (2025) Regenerative viticulture and climate change resilience. Oeno One 59 10.20870/oeno-one.2025.59.1.8089

[10] Oliveira M, Gonçalves I, Soares R, et al (2024) Enhancing vineyard resilience: evaluating sustainable practices in the Douro demarcated region. In: OIV 2024. International Viticulture and Enology Society. 10.58233/3B5KXMEA

[11] Serrano AS, Chacón-Vozmediano JL, Martínez-Gascueña J et al (2024) Could varieties genetically related to Tempranillo behave better than it under drought conditions? Sci Hortic 331:113157. 10.1016/j.scienta.2024.113157

[12] Labarga D, Mairata A, Puelles M et al (2023) The rootstock genotypes determine drought tolerance by regulating aquaporin expression at the transcript level and phytohormone balance. Plants 12(4):718. 10.3390/plants12040718 36840066 10.3390/plants12040718 PMC9961603

[13] Soltani A-A, Khavazi K, Asadi-Rahmani H et al (2010) Plant growth promoting characteristics in some *Flavobacterium* spp. isolated from soils of Iran. J Agric Sci 2(4):106. 10.5539/jas.v2n4p106

[14] Mehlich A (1984) Mehlich 3 soil test extractant: A modification of Mehlich 2 extractant. Commun Soil Sci Plant Anal 15:1409–1416. 10.1080/00103628409367568

[15] Bolyen E, Rideout JR, Dillon MR et al (2019) Reproducible, interactive, scalable and extensible microbiome data science using QIIME 2. Nat Biotechnol 37:852–857. 10.1038/s41587-019-0209-9 31341288 10.1038/s41587-019-0209-9 PMC7015180

[16] Estaki M, Jiang L, Bokulich NA et al (2020) QIIME 2 enables comprehensive end-to-end analysis of diverse microbiome data and comparative studies with publicly available data. Curr Protoc Bioinformatics 70:e100. 10.1002/cpbi.100 32343490 10.1002/cpbi.100 PMC9285460

[17] Callahan BJ, McMurdie PJ, Rosen MJ et al (2016) DADA2: high-resolution sample inference from Illumina amplicon data. Nat Methods 13:581–583. 10.1038/nmeth.3869 27214047 10.1038/nmeth.3869 PMC4927377

[18] Bokulich NA, Kaehler BD, Rideout JR et al (2018) Optimizing taxonomic classification of marker-gene amplicon sequences with QIIME 2’s q2-feature-classifier plugin. Microbiome 6:90. 10.1186/s40168-018-0470-z 29773078 10.1186/s40168-018-0470-z PMC5956843

[19] Quast C, Pruesse E, Yilmaz P et al (2013) The SILVA ribosomal RNA gene database project: improved data processing and web-based tools. Nucleic Acids Res 41(1):590–596. 10.1093/nar/gks1219 10.1093/nar/gks1219 PMC3531112 23193283

[20] Oksanen, Jari & Simpson, Gavin & Blanchet, F. Guillaume & Kindt, Roeland & Legendre, Pierre & Minchin, Peter & hara, R & Solymos, Peter & STEVENS, HENRY & Szöcs, Eduard & Wagner, Helene & Barbour, Matt & Bedward, Michael & Bolker, Ben & Borcard, Daniel & Carvalho, Gustavo & Chirico, Michael & De Cáceres, Miquel & Durand, Sesbastien & Weedon, James. (2022). vegan community ecology package version 2.6–2 April 2022.

[21] Wickham H (2007) Reshaping data with the reshape package. J Stat Soft 21(12):1–20. 10.18637/jss.v021.i12

[22] Wickham H (2016) ggplot2: Elegant Graphics for Data Analysis. J Stat Softw 77 10.1007/978-3-319-24277-4

[23] Wickham H, François R, Henry L, Müller K, Vaughan D (2026). dplyr: A Grammar of Data Manipulation. R package version 1.2.1, https://dplyr.tidyverse.org.

[24] Wickham H, Averick M, Bryan J et al (2019) Welcome to the Tidyverse. J Open Source Softw 4:1686. 10.21105/joss.01686

[25] Ho A, Di Lonardo DP, Bodelier PLE (2017) Revisiting life strategy concepts in environmental microbial ecology. FEMS Microbiol Ecol 93:fix006. 10.1093/femsec/fix006 10.1093/femsec/fix006 28115400

[26] Fierer N, Bradford MA, Jackson RB (2007) Toward an ecological classification of soil bacteria. Ecology 88(6):1354–1364. 10.1890/05-1839 17601128 10.1890/05-1839

[27] R Core Team (2024) R: A language and environment for statistical computing. R Foundation for Statistical Computing, Vienna, Austria

[28] Kanehisa M, Furumichi M, Sato Y et al (2023) KEGG for taxonomy-based analysis of pathways and genomes. Nucleic Acids Res 51:D587–D592. 10.1093/nar/gkac963 36300620 10.1093/nar/gkac963 PMC9825424

[29] Kanehisa M, Goto S (2000) KEGG: Kyoto encyclopedia of genes and genomes. Nucleic Acids Res 28(1):27–30. 10.1093/nar/28.1.27 10592173 10.1093/nar/28.1.27 PMC102409

[30] Douglas GM, Maffei VJ, Zaneveld JR et al (2020) PICRUSt2 for prediction of metagenome functions. Nat Biotechnol 38:685–688. 10.1038/s41587-020-0548-6 32483366 10.1038/s41587-020-0548-6 PMC7365738

[31] Callahan BJ, Sankaran K, Fukuyama JA et al (2016) Bioconductor workflow for microbiome data analysis: from raw reads to community analyses. F1000Res. 10.12688/F1000RESEARCH.8986.1 27508062 10.12688/f1000research.8986.1 PMC4955027

[32] Fanai A, Bohia B, Lalremruati F, Lalhriatpuii N, Lalmuanpuii R, Singh PK (2024) Plant growth promoting bacteria (PGPB)-induced plant adaptations to stresses: an updated review. PeerJ 12:e17882. 10.7717/peerj.17882 39184384 10.7717/peerj.17882 PMC11344539

[33] Giannelli G, Bisceglie F, Pelosi G et al (2022) Phyto-beneficial traits of rhizosphere bacteria: in vitro exploration of plant growth promoting and phytopathogen biocontrol ability of selected strains isolated from harsh environments. Plants. 10.3390/plants11020230 35050118 10.3390/plants11020230 PMC8779669

[34] İpek, M., Eşitken, A. (2017). The Actions of PGPR on Micronutrient Availability in Soil and Plant Under Calcareous Soil Conditions: An Evaluation over Fe Nutrition. In: Singh, D., Singh, H., Prabha, R. (eds) Plant-Microbe Interactions in Agro-Ecological Perspectives. Springer, Singapore. 10.1007/978-981-10-6593-4_4

[35] Coller E, Cestaro A, Zanzotti R et al (2019) Microbiome of vineyard soils is shaped by geography and management. Microbiome 7(1):140. 10.1186/s40168-019-0758-7 31699155 10.1186/s40168-019-0758-7 PMC6839268

[36] Griggs RG, Steenwerth KL, Mills DA et al (2021) Sources and assembly of microbial communities in vineyards as a functional component of winegrowing. Front Microbiol 12:673810. 10.3389/fmicb.2021.673810 33927711 10.3389/fmicb.2021.673810 PMC8076609

[37] Silva V, Brito I, Alexandre A (2025) The vineyard microbiome: how climate and the main edaphic factors shape microbial communities. Microorganisms 13(5):1092. 10.3390/microorganisms13051092 40431264 10.3390/microorganisms13051092 PMC12114118

[38] Zhou J, Cavagnaro TR, De Bei R et al (2021) Wine terroir and the soil bacteria: an amplicon sequencing–based assessment of the Barossa Valley and its sub-regions. Front Microbiol. 10.3389/fmicb.2020.597944 33488543 10.3389/fmicb.2020.597944 PMC7817890

[39] Fierer N (2017) Embracing the unknown: disentangling the complexities of the soil microbiome. Nat Rev Microbiol 15:579–590. 10.1038/nrmicro.2017.87 28824177 10.1038/nrmicro.2017.87

[40] Viñas M, Marull J, Guivernau M et al (2025) Impact of organic and conventional agricultural management on subsurface soil microbiota in Mediterranean vineyards. Agronomy 15:2001. 10.3390/agronomy15082001

[41] Tecon R, Or D (2017) Biophysical processes supporting the diversity of microbial life in soil. FEMS Microbiol Rev 41:599–623. 10.1093/femsre/fux039 28961933 10.1093/femsre/fux039 PMC5812502

[42] Bastida F, Eldridge DJ, García C et al (2021) Soil microbial diversity–biomass relationships are driven by soil carbon content across global biomes. ISME J 15:2081–2091. 10.1038/s41396-021-00906-0 33564112 10.1038/s41396-021-00906-0 PMC8245509

[43] Schimel JP, Schaeffer SM (2012) Microbial control over carbon cycling in soil. Front Microbiol 3:348. 10.3389/fmicb.2012.00348 23055998 10.3389/fmicb.2012.00348 PMC3458434

[44] Biddoccu M, Ferraris S, Opsi F, Cavallo E (2016) Long-term monitoring of soil management effects on runoff and soil erosion in sloping vineyards in Alto Monferrato (North-West Italy). Soil Tillage Res 155:176–189. 10.1016/j.still.2015.07.005

[45] Darouich H, Ramos TB, Pereira LS et al (2022) Water use and soil water balance of Mediterranean vineyards under rainfed and drip irrigation management: evapotranspiration partition and soil management modelling for resource conservation. Water 14:554. 10.3390/w14040554

[46] Novara A, Gristina L, Saladino SS et al (2011) Soil erosion assessment on tillage and alternative soil managements in a Sicilian vineyard. Soil Tillage Res 117:140–147. 10.1016/j.still.2011.09.007

[47] Hartmann M, Brunner I, Hagedorn F et al (2017) A decade of irrigation transforms the soil microbiome of a semi-arid pine forest. Mol Ecol 26:1190–1206. 10.1111/mec.13995 28028891 10.1111/mec.13995

[48] Ogola HJO, Selvarajan R, Tekere M (2021) Local geomorphological gradients and land use patterns play key role on the soil bacterial community diversity and dynamics in the highly endemic indigenous Afrotemperate coastal scarp forest biome. Front Microbiol 12:592725. 10.3389/fmicb.2021.592725 33716998 10.3389/fmicb.2021.592725 PMC7943610

[49] Tripathi BM, Moroenyane I, Sherman C et al (2017) Trends in taxonomic and functional composition of soil microbiome along a precipitation gradient in Israel. Microb Ecol 74:168–176. 10.1007/s00248-017-0931-0 28074247 10.1007/s00248-017-0931-0

[50] Fierer N, Jackson RB (2006) The diversity and biogeography of soil bacterial communities. Proc Natl Acad Sci U S A 103(3):626–631. 10.1073/pnas.0507535103 16407148 10.1073/pnas.0507535103 PMC1334650

[51] Malik AA, Puissant J, Buckeridge KM et al (2018) Land use driven change in soil pH affects microbial carbon cycling processes. Nat Commun 9(1):3591. 10.1038/s41467-018-05980-1 30181597 10.1038/s41467-018-05980-1 PMC6123395

[52] Naz M, Dai Z, Hussain S et al (2022) The soil pH and heavy metals revealed their impact on soil microbial community. Journal of Environmental Management 321:115770. 10.1016/j.jenvman.2022.115770 36104873 10.1016/j.jenvman.2022.115770

[53] Ahmed E, Holmström SJM (2014) Siderophores in environmental research: roles and applications. Microb Biotechnol 7:196–208. 10.1111/1751-7915.12117 24576157 10.1111/1751-7915.12117 PMC3992016

[54] Colombo C, Palumbo G, He JZ et al (2014) Review on iron availability in soil: interaction of Fe minerals, plants, and microbes. J Soils Sediments 14:538–548. 10.1007/s11368-013-0814-z

[55] Lauber CL, Hamady M, Knight R, Fierer N (2009) Pyrosequencing-based assessment of soil pH as a predictor of soil bacterial community structure at the continental scale. Appl Environ Microbiol 75:5111–5120. 10.1128/AEM.00335-09 19502440 10.1128/AEM.00335-09 PMC2725504

[56] Ricci L, Moreau J-L, Petrova N et al (2021) Soil pH and its effect on nutrient availability in acidic and alkaline soils. J Soil Fut Res 2(2):46–56

[57] Hamdali H, Bouizgarne B, Hafidi M et al (2008) Screening for rock phosphate solubilizing actinomycetes from Moroccan phosphate mines. Appl Soil Ecol 38:12–19. 10.1016/j.apsoil.2007.08.007

[58] Jiang ZM, Mou T, Sun Y et al (2023) Environmental distribution and genomic characteristics of *Solirubrobacter*, with proposal of two novel species. Front Microbiol 14:1267771. 10.3389/fmicb.2023.1267771 38107860 10.3389/fmicb.2023.1267771 PMC10722151

[59] Penn CJ, Camberato JJ (2019) A critical review on soil chemical processes that control how soil ph affects phosphorus availability to plants. Agriculture 9(6):120. 10.3390/agriculture9060120

[60] Trivedi P, Leach JE, Tringe SG et al (2020) Plant–microbiome interactions: from community assembly to plant health. Nat Rev Microbiol 18:607–621. 10.1038/s41579-020-0412-1 32788714 10.1038/s41579-020-0412-1

[61] Canfora L, Vendramin E, Felici B et al (2018) Vineyard microbiome variations during different fertilisation practices revealed by 16s rRNA gene sequencing. Appl Soil Ecol 125:71–80. 10.1016/j.apsoil.2017.12.019

[62] Steiner M, Falquet L, Fragnière AL et al (2024) Effects of pesticides on soil bacterial, fungal and protist communities, soil functions and grape quality in vineyards. Ecol Solut Evid 5(2):e12327. 10.1002/2688-8319.12327

[63] Zhou W, Dong J, Ding D et al (2021) Rhizosphere microbiome dynamics in tropical seagrass under short-term inorganic nitrogen fertilization. Environ Sci Pollut Res Int 28(15):19021–33. 10.1007/s11356-020-12048-5 33394400 10.1007/s11356-020-12048-5

[64] Tedesco S, Erban A, Gupta S et al (2021) The impact of metabolic scion–rootstock interactions in different grapevine tissues and phloem exudates. Metabolites 11(6):349. 10.3390/metabo11060349 34070718 10.3390/metabo11060349 PMC8228596

[65] Compant S, Clément C, Sessitsch A (2010) Plant growth-promoting bacteria in the rhizo- and endosphere of plants: their role, colonization, mechanisms involved and prospects for utilization. Soil Biol Biochem 42:669–678. 10.1016/j.soilbio.2009.11.024

[66] Labarga D, Mairata A, Puelles M et al (2025) Rootstocks and drought stress impact the composition and functionality of grapevine rhizosphere bacterial microbiota. Microbiol Res. 10.1016/j.micres.2025.128073 39864304 10.1016/j.micres.2025.128073

[67] Seo H, Kim JH, Lee SM, Lee SW (2024) The plant-associated Flavobacterium: a hidden helper for improving plant health. Plant Pathol J 40:251–260. 10.5423/ppj.rw.01.2024.0019 38835296 10.5423/PPJ.RW.01.2024.0019 PMC11162857

[68] Zhang C, Cai K, Li M et al (2022) Plant-growth-promoting potential of PGPE isolated from *Dactylis glomerata* L. Microorganisms. 10.3390/microorganisms10040731 35456782 10.3390/microorganisms10040731 PMC9032031

[69] Chen L, Li K, Shang J et al (2021) Plant growth–promoting bacteria improve maize growth through reshaping the rhizobacterial community in low-nitrogen and low-phosphorus soil. Biol Fertil Soils 57:1075–1088. 10.1007/s00374-021-01598-6

[70] Mehmood N, Saeed M, Zafarullah S et al (2023) Multifaceted impacts of plant-beneficial *Pseudomonas spp.* in managing various plant diseases and crop yield improvement. ACS Omega 8:22296–22315. 10.1021/acsomega.3c00870 37396244 10.1021/acsomega.3c00870 PMC10308577

[71] Berendsen RL, Pieterse CMJ, Bakker PAHM (2012) The rhizosphere microbiome and plant health. Trends Plant Sci 17:478–486. 10.1016/j.tplants.2012.04.001 22564542 10.1016/j.tplants.2012.04.001

[72] Norris N, Levine NM, Fernandez VI, Stocker R (2021) Mechanistic model of nutrient uptake explains dichotomy between marine oligotrophic and copiotrophic bacteria. PLoS Comput Biol 17:e1009023. 10.1371/journal.pcbi.1009023 34010286 10.1371/journal.pcbi.1009023 PMC8168909

[73] Kuzyakov Y, Blagodatskaya E (2015) Microbial hotspots and hot moments in soil: concept & review. Soil Biol Biochem 83:184–199. 10.1016/j.soilbio.2015.01.025

[74] Naylor D, Coleman-Derr D (2018) Drought stress and root-associated bacterial communities. Front Plant Sci 8:2223. 10.3389/fpls.2017.02223 29375600 10.3389/fpls.2017.02223 PMC5767233

[75] Williams A, de Vries FT (2020) Plant root exudation under drought: implications for ecosystem functioning. New Phytol 225:1899–1905. 10.1111/nph.16223 31571220 10.1111/nph.16223

[76] Zhalnina K, Louie KB, Hao Z et al (2018) Dynamic root exudate chemistry and microbial substrate preferences drive patterns in rhizosphere microbial community assembly. Nat Microbiol 3:470–480. 10.1038/s41564-018-0129-3 29556109 10.1038/s41564-018-0129-3

[77] Nuccio EE, Starr E, Karaoz U et al (2020) Niche differentiation is spatially and temporally regulated in the rhizosphere. ISME J 14:999–1014. 10.1038/s41396-019-0582-x 31953507 10.1038/s41396-019-0582-x PMC7082339

[78] Gómez JA, Guzmán MG, Giráldez JV, Fereres E (2009) The influence of cover crops and tillage on water and sediment yield, and on nutrient, and organic matter losses in an olive orchard on a sandy loam soil. Soil Tillage Res 106:137–144. 10.1016/j.still.2009.04.008

[79] Zhang M, Song X, Wu X et al (2024) Microbial regulation of aggregate stability and carbon sequestration under long-term conservation tillage and nitrogen application. Sust Prod Consum 44:74–86. 10.1016/j.spc.2023.11.022

[80] Fraga H, Costa R, Santos J (2018) Modelling the Terroir of the Douro Demarcated Region, Portugal. In: E3S Web of Conferences. EDP Sciences. 10.1051/e3sconf/20185002009

[81] Belda I, Zarraonaindia I, Perisin M, et al (2017) From vineyard soil to wine fermentation: Microbiome approximations to explain the “terroir” Concept. Front Microbiol 8:821. 10.3389/fmicb.2017.00821 10.3389/fmicb.2017.00821 PMC5420814 28533770

[82] Deyett E, Rolshausen PE (2020) Endophytic microbial assemblage in grapevine. FEMS Microbiol Ecol 96:5. 10.1093/femsec/fiaa053 10.1093/femsec/fiaa053 32196076

[83] Xue PP, Carrillo Y, Pino V, et al (2018) Soil properties drive microbial community structure in a large scale transect in south eastern australia. Sci Rep 8:11725. 10.1038/s41598-018-30005-8 10.1038/s41598-018-30005-8 PMC6078944 30082740

[84] Delroy B, Zhang HY, Bissett A, Powell JR (2024) Divergent responses between lineages of arbuscular mycorrhizal fungi to soil phosphorus and nitrogen availability. Pedobiologia (Jena) 103:150934. 10.1016/j.pedobi.2024.150934

[85] Costa JM, Egipto R, Aguiar FC, et al (2023) The role of soil temperature in mediterranean vineyards in a climate change context. Front Plant Sci 14. 10.3389/fpls.2023.1145137 10.3389/fpls.2023.1145137 PMC10205021 37229125

[86] Berlanas C, Berbegal M, Elena G, et al (2019) The fungal and bacterial rhizosphere microbiome associated with grapevine rootstock genotypes in mature and young vineyards. Front Microbiol 10:1142. 10.3389/fmicb.2019.01142 10.3389/fmicb.2019.01142 PMC6538693 31178845

[87] Aguilar MO, Alvarez F, Medeot D, et al (2021) Screening of epiphytic rhizosphere-associated bacteria in argentinian malbec and cabernet-sauvignon vineyards for potential use as biological fertilisers and pathogen-control agents. Oeno One 55:145–157. 10.20870/oeno-one.2021.55.4.4655

[88] Dries L, Ratering S, Bussotti S, et al (2023) The transcriptionally active bacterial communities of grapevine rhizosphere in dependence on rootstock and scion variety. Oeno One 57:86–97. 10.20870/oeno-one.2023.57.3.5547

[89] Samad A, Trognitz F, Compant S, et al (2017) Shared and host-specific microbiome diversity and functioning of grapevine and accompanying weed plants. Environ Microbiol 19:1407–1424. 10.1111/1462-2920.13618 10.1111/1462-2920.13618 27871147

[90] D’Amico F, Candela M, Turroni S, et al (2018) The rootstock regulates microbiome diversity in root and rhizosphere compartments of vitis vinifera cultivar lambrusco. Front Microbiol 9:22–40. 10.3389/fmicb.2018.02240 10.3389/fmicb.2018.02240 PMC6169447 30319569

[91] OIV (2024) World Wine Production Outlook OIV First Estimates. Internation Organization of Vine and Wine, Paris. https://www.oiv.int/sites/default/files/2024-11/OIV_2024_World_Wine_Production_Outlook.pdf

