# Supplementary material for: Influence of Environment and Rootstock On the Rhizosphere Bacterial Communities in Four Vineyards of the Douro Demarcated Region
Source: Microb Ecol. 2026 Jun 11;89:159. doi: 10.1007/s00248-026-02798-z (PMC13481489; doi:10.1007/s00248-026-02798-z)
Supplement: Supplementary file 1 [file 248_2026_2798_MOESM1_ESM.docx]

***Electronic Supplementary Material***

**Journal:** Microbial Ecology

**Article Title:** Environmental and genetic traits modulate the rhizosphere microbiome of vineyards of the Douro Demarcated Region

**Authors:** João Prada, Leandro Pereira-Dias, João A. Santos, Conceição Santos

**Corresponding author:** João Prada (joao.prada@fc.up.pt)

**Supplementary Table S1**

|  | **pH (H2O)** | **pH (CaCl2)** | **Organic matter (%)** | **C/N** | **P (mg/kg P2O5)** | **K (mg/kg K2O)** | **Ca (mg/kg CaO)** | **Mg (mg/kg MgO)** | **Fe (mg/kg Fe)** | **Mn (mg/kg Mn)** | **Cu (mg/kg Cu)** | **S (mg/kg S)** |
| --- | --- | --- | --- | --- | --- | --- | --- | --- | --- | --- | --- | --- |
| **Cavernelho** | 7.2 | 6.7 | 1.63 | 13 | 87.8 | 471 | 1832 | 152 | 89.7 | 66 | 17.6 | 9.48 |
|  | 6.4 | 5.8 | 1 | 11 | 341 | 442 | 1090 | 205 | 58 | 22.9 | 17.3 | 8.5 |
|  | 6.7 | 6 | 1.05 | 11 | 99.7 | 367 | 974 | 166 | 81 | 22.1 | 18.7 | 8.5 |
| **Seixo** | 6.9 | 6.3 | 1 | 9 | 67.1 | 39.8 | 2841 | 765 | 98.5 | 23.9 | 8.93 | 8.5 |
|  | 7.1 | 6.5 | 1 | 9.4 | 183 | 30.5 | 3041 | 882 | 116 | 31.9 | 8.6 | 10.5 |
|  | 6.5 | 6.1 | 1 | 9.6 | 112 | 55.5 | 2788 | 616 | 147 | 13.1 | 23.6 | 11.9 |
| **Belém** | 6 | 5.4 | 1 | 11 | 102 | 55.2 | 1787 | 396 | 119 | 21.6 | 0.478 | 8.5 |
|  | 6.4 | 5.3 | 1.03 | 12 | 75.9 | 43.2 | 1676 | 388 | 103 | 17.6 | 0.615 | 8.5 |
|  | 5.7 | 4.5 | 1 | 7.9 | 47 | 45.6 | 1020 | 348 | 91.7 | 12.7 | 0.4 | 8.5 |
| **Ervamoira** | 7.2 | 6.4 | 1.02 | 9.9 | 100 | 31.1 | 3130 | 654 | 164 | 75.1 | 4.41 | 8.5 |
|  | 7.3 | 6.5 | 1 | 9.1 | 101 | 34.3 | 3805 | 750 | 147 | 60.7 | 3.98 | 8.5 |
|  | 7.2 | 6.5 | 1.28 | 11 | 82.6 | 35.4 | 3855 | 809 | 134 | 55.1 | 4.53 | 8.5 |

**Supplementary Table S2**

**Supplementary Table S 1:** Relative abundance (%) of the top 20 most abundant bacterial genera in the rhizosphere baseline (Summer 2022)

| TOP 20 GENERA SUMMER 2022 | | | | |
| --- | --- | --- | --- | --- |
| Genera | BL | CV | EM | SX |
| Acidibacter | 0.27 | 0.65 | 0.88 | 0.6 |
| Bacillus | 3.13 | 2.27 | 2.87 | 1.55 |
| Bradyrhizobium | 1.85 | 1.04 | 1.52 | 2.34 |
| Bryobacter | 0.74 | 0.49 | 0.73 | 1.04 |
| Candidatus_Solibacter | 0.79 | 0.56 | 0.46 | 1.36 |
| Candidatus_Udaeobacter | 0.29 | 0.42 | 0.26 | 3.64 |
| Conexibacter | 1.45 | 0.4 | 1.81 | 2.42 |
| Dongia | 0.25 | 0.75 | 0.82 | 1.11 |
| Ellin6067 | 0.8 | 0.99 | 0.5 | 0.5 |
| Gaiella | 0.41 | 1.33 | 2.26 | 2.67 |
| Gemmatimonas | 0.9 | 0.5 | 0.51 | 0.64 |
| Haliangium | 0.56 | 0.97 | 0.73 | 0.73 |
| Less than 1% | 25.21 | 22.52 | 20.25 | 15.59 |
| MND1 | 0.51 | 1.39 | 0.72 | 0.62 |
| Mycobacterium | 0.61 | 0.58 | 0.76 | 0.75 |
| Nocardioides | 0.88 | 0.93 | 1.37 | 0.6 |
| Other | 1.18 | 0 | 1.15 | 0 |
| RB41 | 0.45 | 2.05 | 1 | 1.21 |
| Solirubrobacter | 0.69 | 1.83 | 4.18 | 3.01 |
| Sphingomonas | 2.02 | 1.21 | 1.75 | 1.5 |
| Streptomyces | 1.1 | 0.87 | 3.88 | 1.86 |
| Subgroup_10 | 1.27 | 0.67 | 0.65 | 0.84 |
| Unassigned | 54.6 | 57.59 | 50.94 | 55.42 |

**Supplementary Table S3**

**Supplementary Table S 3:** Kruskal-Wallis rank-sum results of the non-parametrics tests in copiotrophic vs. oligotrophic bacteria

| Strategy | Group1 | Group2 | p-value | Significance |
| --- | --- | --- | --- | --- |
| K-Strategists | CV | EM | 0.057 | No |
| K-Strategists | SX | EM | 0.114 | No |
| K-Strategists | BL | SX | 0.200 | No |
| K-Strategists | CV | BL | 0.343 | No |
| K-Strategists | CV | SX | 1.000 | No |
| K-Strategists | BL | EM | 1.000 | No |
| r-Strategists | CV | EM | 0.029 | Yes |
| r-Strategists | CV | SX | 0.057 | No |
| r-Strategists | BL | EM | 0.057 | No |
| r-Strategists | CV | BL | 0.200 | No |
| r-Strategists | BL | SX | 0.343 | No |
| r-Strategists | SX | EM | 0.886 | No |

**Supplementary Fig. S1**

**Supplementary Fig. 1:** Rarefaction curves of the rhizosphere microbiome across the four vineyard sites (baseline). The red dashed line indicates the chosen rarefaction depth of 25,000 reads, at which all sample curves reached a clear asymptote, demonstrating sufficient sequencing depth to capture community diversity.
